# Embitterment and metacognition in obsessive–compulsive disorder

**DOI:** 10.1186/s12888-023-04642-x

**Published:** 2023-03-08

**Authors:** Paraskevi Mavrogiorgou, Sarah Becker, Sie-In Lee-Grimm, Georg Juckel

**Affiliations:** 1 Department of Psychiatry, Psychotherapy and Preventive Medicine, LWL-University Hospital of Ruhr-University Bochum, Bochum, Germany; 2 Department of Psychiatry, Ruhr-University Bochum, LWL-University Hospital, Alexandrinenstr.1, 44791 Bochum, Germany

**Keywords:** Embitterment, Post-traumatic embitterment disorder (PTED), Metacognition, Obsessive–compulsive disorder

## Abstract

**Objective:**

Embitterment is a persistent emotion that is known to everybody in reaction to injustice and being let down, associated with feelings of helplessness and hopelessness. People with psychiatric disorders can develop bitterness, which is to be understood as a form of reactive embitterment to the illness. The aim of this explorative study was to investigate the occurrence of embitterment in obsessive–compulsive patients compared to healthy volunteers and in the context of their metacognitions and other biographical and clinical characteristics.

**Method:**

Following a semi-structured diagnostic interview, a number of measures were administered to 31 patients with obsessive–compulsive disorder (OCD) [ICD-10 F42.X: mean age 35.2 (SD = 10.7) years] and 31 healthy volunteers [mean age 39.1 (SD = 15.0) years]. These measures included the Post-Traumatic Embitterment Disorder questionaire (PTEDq) for measuring embitterment, the Yale-Brown Obsessive–Compulsive Scale, the Metacognition Questionnaire and other psychometric questionnaires such as the Beck Depression Inventory and the State-Trait Anxiety Inventory.

**Results:**

Patients with OCD scored more than three times higher (mean = 2.0, SD = 1.1) than the healthy participants in the PTEDq (mean = 0.6, SD = 0.8; *p* < 0.001), but the cut-off of < 2.5 for a clinically relevant embitterment disorder was not reached. Dysfunctionally distorted metacognition (MCQ-30), which is a consistent finding in OCD, as well as a generally high degree of clinical impairment were significantly cor related to the degree of embitterment.

**Conclusion:**

Our findings suggest that embitterment as measured by PTEDq is important in patients with OCD, who are characterized by metacognitive distortions with an injustice of fate as well as a mortification of their self-image. In future, it would be necessary to screen patients with OCD not only for depressive symptoms but also specifically for feelings of embitterment in order to be able to initiate appropriate psychotherapeutic measures at an early stage.

## Introduction

In the course of a lifetime, everyone experiences interpersonal conflicts, injuries, slights, injustice or unfairness and associated negative feelings of resentment, anger, bitterness and rage to varying degrees. Feelings of anger and revenge after injuries are considered appropriate and adaptive psychological reactions for short-term coping, but they can lead to post-traumatic embitterment disorder [1, 2] if they are prolonged and severe. Embitterment can develop as a result of extraordinary, but quite normal, stresses that are personally experienced as mortifying, degrading and subjectively as unjust [2]. Several factors are important for the development and manifestation of embitterment: that the patient considers the event to be causal for his/her psychological state; that the patient has subjectively experienced the event as unjust or as humiliation; that the patient’s reaction to the event includes feelings of bitterness, anger and helplessness; and that the patient reacts with emotional arousal when reminded of the event [1]. Persisting in a passive victim role, a defiant, long-lasting emotion of bitterness and insistence on justice and unforgiveness can lead to a psychiatric symptomatology that is relevant to PTED, which often appears depressive and is misjudged as a depressive disorder. People with psychiatric illnesses can also develop bitterness, which is to be understood as a form of ‘reactive embitterment’ that develops secondarily as a comorbidity in the context of an existing mental illness. In a study of inpatients with mental disturbances (*n* = 1032), Linden and Maercker [3] were able to show that reactive embitterment was a frequent accompanying symptom in a variety of psychiatric disorders. According to the diagnostic construct of PTED, an existing mental illness is considered an exclusion criterion for diagnosis but it is still possible that people with mental disorders have significantly elevated levels of embitterment. This may be because their illness increases their susceptibility to pathologically increased bitterness or because they experience critical, unjust or upsetting life events more frequently due to their disorder [4].

The fact of suffering from a mental disorder also represents a critical and, above all, long-lasting life event for many affected persons due to the social stigmatization, the pressure to suffer and the possible restrictions in coping with everyday life, which can cause an intense feeling of bitterness. This is to be regarded as critical, especially against the background that a defiant, long-lasting bitterness in the sense of a ‘vicious circle’ can cause worsening and perpetuation of the disease-related symptoms. The patients are then no longer able to recognize that the actual problem is no longer the past blow of fate but the continuing embitterment and their own victim role [5]. This potentially dangerous mixture of feelings makes those affected seek professional help less often and makes the success of therapy more difficult due to the associated resigned, sometimes aggressive, basic attitude and thus the development of a new perspective on life [5]. However, embitterment (ranging from embitterment syndrome up to PTED) is also considered a critical life event in the context of other mental and also somatic illnesses as a reaction to them [6]. However, the extent to which embitterment may represent a psychologically relevant dimension in obsessive–compulsive disorder (OCD) has not yet been examined in detail.

Phenomenologically characteristic of OCD is the occurrence of repetitive, unpleasant obsessive thoughts and/or compulsive actions, which are accompanied by a high level of suffering on the part of the person affected (often also on the part of the relatives) and with significant impairment of everyday life. Metacognition is an awareness of one's thought processes and an understanding of the patterns behind them. Metacognition can take many forms, such as reflecting on one's ways of thinking and knowing when and how to use particular strategies for problem-solving. In several studies, a pronounced impairment of metacognition in the form of increased negative evaluation and increased need for control of their thinking could be demonstrated in OCD patients. In this context, it has also been shown that patients are often characterized by a negative attitude towards their thoughts and thinking style, which usually correlates with the severity of OCD [7, 8]. Knowledge of one’s own thought processes was also found to be increased in patients with OCD, in the form of generally increased attention to one’s own process [9].

This increased focus on one’s own thinking and thought processes as well as unwanted automatic thoughts lead, among other things, to the well-known symptomatology in OCD patients: increased rigidity, lack of cognitive flexibility, lack of trust in one’s own memory and thus an increased need for control [10]. According to Wells’ theory, the genesis of compulsions is thought to be primarily due to thinking in the ‘object mode’ of compulsive patientsWhereas mentally healthy people usually perceive their own thoughts merely as an evaluation of reality, which enables them to better adapt to reality, compulsive patients perceive their own thoughts as objective facts and a direct image of reality. Within the framework of this thought–action fusion, patterns of conviction result in which an imposing thought is judged to be just as morally reprehensible as the actual action [9]. Thought action fused moral beliefs can lead to obsessive thoughts being interpreted in such a way that a sinful thought could be the implication of one’s own morally reprehensible character. However, such belief patterns also appear to be significantly involved in the development of an emotion of embitterment. Linden [1], for example, postulates in the context of his concept of embitterment that it results from a profound mortification and violation of one’s own innate and strongly internalized basic assumptions acquired through early childhood imprinting. These basic assumptions, which include political, relegious and moral convictions, fundamental ideas and values about one’s own self and personal goals in life, are needed to steer coherent behaviour throughout the entire life cycle. This ultimately makes them extraordinarily resistant to external influences and changes. If these rigid patterns of conviction are now threatened or devalued, this can cause either active opposition or passive behaviour in the form of an immense resentment effect [5].

OCD can be seen by the patients as an injustice of fate and may also result in many injustices and downgradings in everyday life, which should result in embitterment emotions and can cause a 'vicious circle' by fostering dysfunctional illness behavior. Since there is no special scale for the measurement of the emotion of embitterment, we and other Gao et al. [11] had to use the questionaire for post-traumatic embitterment disorder (PTEDq), as it was developed by M. Linden. We only use this scale to assess embitterment as symptom. None of the subjects studied have reached the cut-off for the embitterment disorder. In addition we have not assumed to find the embitternment disorder in OCD patients. PTEDq is mostly related to measurements of emotions such as embitterment, sadness and disappointment. This scale is not related to cognitive parameters such as metacognition, psychopathological beliefes or some rumination processes. Against this background, it seems plausible that the affected patients could develop a disturbance pattern with bitterness as part of their OCD. Whether this is actually the case has not been investigated to date. The aim of this explorative study was therefore to investigate whether patients with OCD have an embitterment as an emotion compared to healthy subjects. In addition, the correlation of bitterness with other biographical-clinical and psychometric characteristics, and here especially the metacognitive characteristics, was to be examined in more detail.

## Material and methods

### Participants

A total of 62 participants (38 women and 24 men) were examined in this study. Of these, 31 patients fulfilled the criteria of OCD [ICD-10 F42.X: mean age 35.2 (SD = 10.7) years] and 31 subjects formed the healthy control group [mean age 39.1 (SD = 15.0) years]. A detailed description of the groups can be found in Table 1.

**Table 1:** Sociodemographic and clinical characteristics

Trait	OCD *N* = 31	HC *N* = 31	*p*-value*
**Age**, mean (SD), years	38,7 (11,9)	39,6 (13,1)	n. s
**Gende**r			
Female n,	19 (61,3%)	19 (61,3%)	n. s
Male n,	12 (38,7%)	12 (38,7%)	
**Marital status**			
Single, n	17 (54,8%)	12(38,7%)	
Married, n	9 (29,0%)	19 (61,3%)	*p* = 0.009
Divorced, n	5 (16,1%)	0	
Current Partnership			
- No	13 (41,9%)	7 (22,6%)	n. s
- Yes	18 (58,1%)	24 (77,4%)	
**Graduation**			
High school, n	25 (80,7%)	24 (77,4)	
Junior high school, n	3 (9,7%)	5 (16,1%)	n. s
Low school., n	3 (9,7%)	2 (6,5%)	
**Occupational status**			
Current employment (Including be a student), n	22 (71,0%)	27 (87,1%)	n. s
No current Job,	9 (29,0%)	4 (12,9%)	
**Diagnosis (ICD-10)**			
F42.0, n	5 (16,1%)	/	
F42.2, n	26 (83,9%)		
**Age of onset**			
mean (SD), years	23,2 (9,1)	/	
**Duration of illness**			
mean (SD), years	15,8 (10,8)	/	

^*^x^2^-Test/ t-Test; *n. s.* non-significant, *OCD* Obsessive–compulsive disorder, *HC* Healthy controls

All OCD patients were recruited and examined during their treatment at the Department of Psychiatry (LWL-University Hospital of the Medical Faculty of Ruhr-University Bochum, special outpatient clinic for OCDs). Examination of the healthy volunteers also took place at the LWL-University Hospital Bochum and recruitment was via notices and flyers.

Patients and healthy volunteers aged 18–67 years were included. Further inclusion criteria were a verbal IQ > 70, sufficient German language skills and the ability to give informed consent according to the Helsinki and ICH-GCP declarations. Exclusion criteria for the study were: severe somatic diseases; other mental diseases, such as reduced intelligence (ICD10 F70–F70.9), schizophrenia (ICD10 F20–F20.9) or organic brain disorders (ICD10 F06–F06.9, dependence on illegal drugs); acute suicidal tendencies or behaviour endangering others; and lack of informed consent to participate in the study.

Furthermore, psychopharmacotherapy was not an exclusion criterion for patients with OCD. In this respect, 96.8% of the patients (*n* = 30) received monotherapy, whereby antidepressants from the selective serotonin reuptake inhibitor group [e.g. sertraline (*n* = 21), escitalopram, paroxetine, fluoxetine] but also clomipramine (a tricyclic antidepressant) were predominantly used. Moreover, seven of the patients received a combination treatment (mainly a sedating antipsychotic medication, e.g. promethazine or quetiapine). At the time of inclusion in the study, 12 patients were receiving psychotherapeutic treatment (validation therapy: *n* = 9; deep psychology: *n* = 3). Only five of the patients (16.1%) with OCD had not received psychotherapy at the time of study inclusion, either currently or in the past. A detailed anamnesis was taken from all OCD patients and healthy volunteers in a semi-structured interview (duration 45–60 min). The psychometric characteristics, including shame and guilty proneness, were gathered using various questionnaires.

The study was approved by the local Ethics Committee (No. 20–6883) of the Medical Faculty of Ruhr-University Bochum.

### Main mesure instrument for embitterment and metacognition

The PTEDq is a 19-item self-assessment questionnaire [12]. The core element of the PTEDq is the statement: ‘During the last years there was a severe and negative life event…’; this is followed by single items (example: ‘which is why I avoid certain places or people so as not to be reminded of them’). Answers are given on a Likert scale of 0–4 (0, *does not apply*; 4, *applies completely*). Finally, the scores are added up and divided by the total number of items to form the PTEDq total score. An average total score of ≥ 2.5 indicates a clinically significant intensity of pathological bitterness, meaning that the embitterment disorder can be assumed.

The MCQ-30 [13] is a shortened version of the original 65-item Metacognition Questionnaire and a self-report assessing metacognitive beliefs about the negative consequences of one’s thoughts. The MCQ-30 has five subcales: positive beliefs about worry; negative beliefs about uncontrollability and danger; cognitive confidence; need to control thoughts; and cognitive self-consciousness. Higher scores indicate stronger beliefs. Reliability and validity were well documented for the German version of the MCQ-30 [14]; for example, the internal consistency (Cronbach’s alpha) for the various subscales was between 0.72 and 0.93.

### Psychometric measurements

The Beck Depression Inventory (BDI-II) [15] and Hamilton Depression Scale (HAMD [16]) were used to assess possible depressive symptoms in the participants. The Clinical Global Impression scale (CGI) [17] was used to measure the overall severity of the patients’ mental illness. The Personal and Social Performance (PSP) scale is a valid, reliable and standardized measuring instrument for recording psychosocial functional level [18]. Anxiety was assessed using the State-Trait Anxiety Inventory (STAI-I and STAI-II) [19] and verbal intelligence was assessed using the Multiple Choice Vocabulary Test (MWT, Version B) [20].

Obsessive–compulsive symptoms were assessed in the patient group using the Yale-Brown Obsessive Compulsive Scale (Y-BOCS) checklist and Y-BOCS severity scale [21], whereas healthy subjects completed the Mini-PLUS Interview [22] and the Maudsley Obsessional Compulsive Inventory (MOCI) [23]. All other questionnaires listed, including the Metacognition Questionnaire (MCQ-30) and the Post-Traumatic Embitterment Disorder (PTED) self-rating scale, were used with all the study participants.

### Statistical analyses

Statistical analyses of the data were performed using IBM SPSS Statistics for Windows, Version 26.0 (IBM Corp., Armonk, NY, USA). For a descriptive analysis of the data, frequencies, mean values and standard deviations were calculated. Correlation analyses were carried out using Pearson’s and Spearman’s correlation coefficients. Statistically significant group differences were determined using parametric and/or non-parametric tests (Student *t*-test, χ^2^ test), whereby *p* ≤ 0.05 was considered to be statistically significant and* p* ≤ 0.01 as highly significant.

## Results

### Descriptive sociodemographic and clinical findings

The sociodemographic findings of the study participants and the significant differences found are shown in Table 1.

Almost 10% (*n* = 3, 9.7%) of the OCD patients reported having been neglected by their parents during childhood and about 6% (*n* = 2, 6.5%) also reported physical violence during their upbringing. In comparison, this did not occur in any of the healthy volunteers. Alcohol dependence of the parents was found in 6.4% (*n* = 2) of the patients and 12.9% (*n* = 4) of the controls. With regard to their relationship status, the two groups differed significantly (*p* = 0.009). More than half (*n* = 17, 54.8%) of the OCD patients were single at the time of the survey, whereas this was true for only 12 (38.7%) of the healthy controls. Nine (29.0%) of the patients were married and five (16.1%) had already divorced from their spouse. In contrast, 19 (61.3%) of the comparison subjects were married and none were divorced.

The mean age at first manifestation of obsessive–compulsive symptoms was 23.2 (SD = 9.1, range = 6–42) years. All patients reported a chronic course of their OCD, which had an average duration of 15.8 (SD = 10.8; range = 2–44) years. An average of 8.4 (± 10.1) years elapsed between the onset of OCD and the first psychiatric diagnosis. In 18 of the 31 (58.1%) patients, other psychiatric disorders were diagnosed as comorbidities [14 patients (45.2%) had a depressive disorder and 34% had a social phobia] in addition to OCD as the main diagnosis.

### Embitterment and metacognitions

**Fig. 1:**
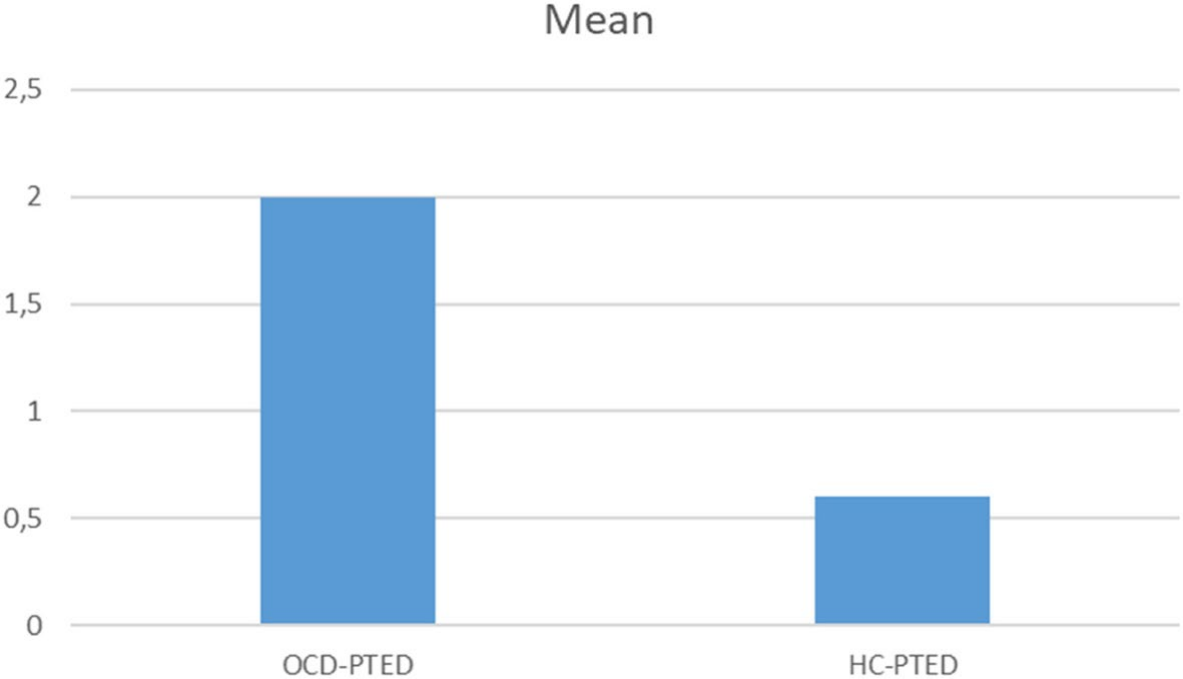
Mean values of PTED-scale of OCD patients and healthy controls. HC = Healthy controls; OCD = Obsessive–compulsive disorder PTED = Posttraumatic embitterment disorder self-rating scale

With regard to embitterment as measured by the PTEDq, the patients with OCD scored more than three times higher (mean = 2.0, SD = 1.1) than the healthy study participants (mean = 0.6, SD = 0.8). This group difference proved to be highly statistically significant at a level of *p* ≤ 0.001 (see also Fig. 1). Analogous to the PTEDq evaluation mask, the cut-off threshold of 2.5 for a clinically relevant embitterment disorder was not reached but the mean sum value of the patients can still be classified as significantly increased.

With an average of just under 70 points of the maximum achievable 120 points of the MCQ-30, the patient group showed a moderately severe metacognitive dysfunction compared to the control group with 46 points. In particular, all the MCQ subscales achieved higher sum scores in the OCD patients and reflected a metacognitive distortion (see Table 2).

**Table 2:** Psychometric characteristics including MCQ-30

**Trait**	OCD *N* = 31	HC *N* = 31	**t***p*-value*
**HAMD**, mean (SD)	10,42 (5,5)	2,13 (2,9)	*p* ≤ 0,001
**BDI**-II, mean (SD)	14,7 (9,4)	4,6 (5,6)	*p* ≤ 0,001
**PSP**, mean (SD)	64,0 (12,3)	93,5 (3,2)	*p* ≤ 0,001
**STAI I,** mean (SD)	47,9 (11,8)	32,0 (7,9)	*p* ≤ 0,001
**STAI II**, mean (SD)	51,9 (11,8)	31,8 (9,4)	*p* ≤ 0,001
**Y-BOCS**, mean (SD)			
obsessions	8,9 (3,9)	/	/
compulsions	6,9 (5,3)		
total	15,9 (7,1)		
**MWT-B**, mean (SD)	107,2 (14,9)	119,3 (13,0)	*p* = 0,001
**MOCI**, mean, (SD)	/	4,2 (2,8)	/
**MCQ-**Positive beliefs mean, (SD)	11,7 (5,0)	8,6 (2,8)	*p* = 0,004
**MCQ-**Danger mean, (SD)	15, 8 (4,7)	8,7 (3,4)	*p* ≤ 0.001
**MCQ-**Cognitive confidence, mean, (SD)	12,1 (3,8)	7,9 (2,4)	*p* ≤ 0.001
**MCQ-**Need to control thoughts mean, (SD)	13,8 (4,4)	9,1 (3,0)	*p* ≤ 0.001
**MCQ-**Cognitive self-consciousness mean, (SD)	16,8 (3,9)	11,4 (2,8)	*p* ≤ 0.001
**MCQ-total** mean, (SD)	69,1 (15,4)	46,1 (8,2)	p ≤ 0.001

*BDI-II* Beck-Depression-Inventory, *HAMD* Hamilton Depression-Scale, *HC* Healthy controls, *MCQ* Metacognitions Questionnaire, *MOCI* Maudsley Obsessional-compulsive Inventory, MWTB- IQ_ Multiple choice vocabulary Intelligence Test, version B; *OCD* Obsessive–compulsive disorder, *PSP* Personal and Social Performance Scale, *STAI* State and Trait Anxiety Inventory, *SD* Standard deviation; *t-Test; Yale-Brown-Obsessive–Compulsive-Scale

### Other psychometric findings

Overall, the patient group had mild depressive symptoms based on the BDI-II and HAMD scores. The corresponding general psychometry values and significant group differences can be seen in Table 2.

With an average total of 64 points on the PSP scale, the OCD patients showed obvious but not pronounced psychosocial difficulties and thus still had sufficient abilities in various areas of their lives (e.g. independence, professional life). In terms of anxiety, they showed slightly to moderately elevated scores on the STAI. With an average total score of just under 16 points on the Y-BOCS severity scale, the patient collective corresponded to the cut-off value for a clear expression of obsessive–compulsive symptoms. In direct comparison, the MOCI did not show any tendency to obsessiveness or obsessive–compulsive symptoms of psychopathological severity in the healthy control group. With regard to verbal intelligence (MWT-B) [24], the groups differed significantly (*p* = 0.001) but both the patients and the mentally healthy participants had a high to very high intelligence level.

In the separate covariance analyses conducted with the covariates HAMD, BDI-II, PSP, STAI-I and STAI-II, MWT-B and MCQ, as well as biographical characteristics such as marital status, partnership and positive family history for mental illness, the significant group difference found in the PTEDq was confirmed (F4/57 = 9.7–24.8; *p* < 0.001), with the influence of BDI-II (*p* ≤ 0.001) and STA-II (*p* = 0.003) being strongest in these models.

### Statistical relationships

Correlations between embitterment and biographical-clinical characteristics were found only for the group of OCD patients. Here, the PTEDq correlated with the presence of a partnership (*r* =  − 0.387, *p* = 0.031), the age at onset of obsessive–compulsive symptoms (*r* =  − 0.479, *p* = 0.006) and the time span between the onset of obsessive–compulsive symptoms and the initial diagnosis (*r* = 0.368, *p* = 0.041).

The correlations of embitterment with the other psychopathological characteristics (including metacognition), which were elicited far more frequently, are shown separately for the OCD patients and the healthy subjects in Table 3. For the patients, there was also a highly significant positive correlation between the severity of the disease state assessed by the CGI and the degree of embitterment (*r* = 0.548, *p* = 0.001).

**Table 3:** Correlations between PTED-Scale and other psychometric characteristics

Variable	**PTED-OCD**	**PTED-HC**
BDI-II	***r* = *0,667; p* ≤ *0.001***	***r* = *0,635; p* ≤ *0.001***
HAM-D	r = 0,315; p = 0,084	***r* = *0,572; p* = *0,001***
PSP	***r* =—*0,463; p* = *0,009***	*r* =—0,263; n. s
STAI-I	***r* = *0,504; p* = *0,004***	***r* = *0,487; p* = *0,005***
STAI-II	***r* = *0,560; p* = *0,001***	***r* = *0,547; p* = *0,001***
MCQ-Positive beliefs	***r* = *0,364; p* = *0,044***	*r* = 0,328; n. s
MCQ-Danger	r = 0,336; p = 0,065	***r* = *0,360; p* = *0,047***
MCQ- Cognitive confidence	r = 0,232; n. s	*r* = 0,050; n. s
MCQ- Need to control thoughts	***r* = *0,455; p* = *0,010***	*r* = 0,052; n. s
MCQ- Cognitive self-consciousness	***r* = *0,362; p* = *0,045***	*r* =—0,223; n. s
MCQ-total	***r* = *0,427; p* = *0,017***	*r *= 0,223; n. s

*BDI-II* Beck-Depression-Inventory, *HAMD* Hamilton Depression-Scale, *HC* Healthy controls, *MCQ* Metacognitions Questionnaire, *n. s.* non-significant, *OCD* Obsessive–compulsive disorder, *PSP* Personal and Social Performance Scale, *PTED* Posttraumatic embitterment disorder self-rating scale, *STAI* State and Trait Anxiety Inventory

Interestingly, the healthy subjects showed a significant correlation between embitterment and compulsiveness measured by the MOCI (*r* = 0.491, *p* = 0.005). In contrast, in OCD patients there was no correlation between the measure of embitterment and either the severity of the OCD or the occurrence of the various subtypes of obsessive–compulsive symptoms, which were assessed using the Y-BOCS checklist.

In order to examine possible influencing factors, and their strength, on the extent of embitterment in more detail, separate multiple linear regression analyses were carried out. The covariates BDI-II (β = 0.630, *p* = 0.003), age at onset (β =  − 0.577, *p* = 0.005) and STAI-II (β = 0.567, *p* = 0.058) proved to be of significant importance for the PTEDq score of the OCD patients. In the healthy subjects, the separate regression analyses also showed depressiveness (BDI-II: β = 0.586, *p* = 0.001) and trait anxiety (STAI-II: β = 0.659, *p* = 0.020) to be significant factors influencing the measure of embitterment.

## Discussion

In this explorative study, it was examined for the first time to what extent the feeling of embitterment, measured by the PTEDq [1], represents a psychopathologically relevant dimension in OCD and in what context it can be classified with other clinical-psychometric and sociodemographic characteristics. The outpatient OCD patients studied here showed a manifest difference in bitterness emotion compared to the mentally healthy volunteers. On average, they scored more than three times as high on the PTEDq but did not reach the cut-off threshold for a clinically highly significant embitterment reaction with illness value in the sense of a possible PTED. The degree of embitterment showed no significant correlation with either the severity or type of obsessive–compulsive symptoms in the affected patients. Interestingly, however, increased compulsiveness as a personality trait was associated with more embitterment in the healthy subjects. In addition, relevant correlations were found between the degree of embitterment and general anxiety as a personality trait, depressive symptoms and a low level of psychosocial functioning.Dysfunctionally distorted metacognition, which is a consistent finding in OCD, as well as a generally high degree of clinical impairment were directly related to the degree of embitterment. Furthermore, correlations were found with the sociodemographic-clinical characteristics of the OCD patients. The younger the patient at onset of the OCD and the longer the duration of the illness, the stronger was the feeling of bitterness. It was also remarkable that a longer period of time between onset of obsessive–compulsive symptoms and receipt of the initial diagnosis was associated with more embitterment. Finally, correlations with the relationship status of the patients could also be demonstrated. Thus, those affected who lived in a partnership reported fewer feelings of bitterness than OCD sufferers without a relationship.

Even if there is no manifest comorbid embvitterment disorder in our OCD patients, their significantly increased PTEDq value can be interpreted as an indication of the presence of one of the four psychopathologically relevant types of embitterment states according to Linden [5]. The presence of a secondary, reactive embitterment as a result of the OCD can be discussed here. According to Linden [5], secondary embitterment is a psychopathologically relevant form of embitterment that can occur in the context of a pre-existing psychiatric or psychosomatic illness [5]. An earlier study by Linden and Maercker [3] on more than 1000 inpatients of a psychosomatic clinic was already able to show that more than 80% of those affected with a personality disorder, 70% of patients with an adjustment disorder, more than 60% of those with depressive disorders and, most recently, almost half of patients with anxiety disorders had PTEDq scores of 2 or more points. The authors concluded that secondary embitterment is a frequent accompanying symptom of many mental illnesses. Although the empirical study by Linden and Maercker [3] did not take a differentiated look at OCD patients, the results are comparable to those of the study presented here. Overall, the role of embitterment in the context of mental illness is still quite little researched, so there are hardly any comparative values from other empirical studies.

It is also unclear how the development of a so-called secondary embitterment reaction can be justified against the background of an OCD. Bitterness typically arises as a reaction to a suffered injustice, a slight or a belittlement that is connected to a violation of central basic assumptions. For the person affected, this results in long-term, negative consequences and, at the same time, there is the impression that nothing can be done to change the injustice that has been done [2]. The psychological foundation of intense bitterness is laid by central basic assumptions. These are culturally and socially transmitted patterns of belief that build up a cognitive reference and value system, with the help of which human experiences, perceptions and behaviour are influenced and controlled [5]. These basic beliefs are learned between the ages of 5 and 20 years [5], so we grow up with them and they have an immense influence on how we think, act and feel. Basic assumptions enable coherent behaviour across the lifespan and need to be understood as strong and important psychology [4].

In the context of a psychiatric illness such as OCD, various breaks with essential basic assumptions can be assumed., The reactive bitterness of the OCD patients could be explained by the fact that being mentally ill is experienced as an injustice of fate and collides with one’s own axiom of justice. The OCD has far-reaching negative consequences for the person affected, such as with regard to social relationships or their professional future, and at the same time it is not immediately possible for the OCD patient to change anything about the current unfortunate situation. The decisive basic assumption violated here by suffering from an OCD is that of the ideology of justice. According to Linden [5], the ideology of justice is one of the most psychologically significant basic human assumptions and describes the tendency to believe that every person gets what he or she deserves, that positive but also negative events can be controlled by means of appropriate behaviour and that meaningful connections exist between a person’s behaviour or character and the blows of fate that befall him or her [5]. This basic assumption is a crucial prerequisite for people to view their environment as just, controllable and predictable, and thus feel safe and secure. On the other hand, this also substantiates the fact that an injustice is experienced like an aggression and that justice ideology plays a prominent role in the context of bitterness [5].For the patients, their OCD could therefore appear as an enormous injustice of fate that they have not deserved and in the face of which they feel helpless and powerless. This injustice suffered can call into question the belief in justice as a whole and weighs so heavily on the individual that assimilation into the existing system of references and values is not possible and accommodation of these schemata to the event is also refused [5]. The OCD sufferer feels frustration, anger, aggression, helplessness and mortification towards his new fate with the OCD symptoms and thus, in summary, the complex feeling of embitterment is combined with repetitive behavior (in the sense of the compulsive repetition of thoughts of bitterness).

This,, and the metacognitve self beliefs can lead to frustration, lack of self-confidence and lack of self-awareness, which can lead to frustration, hopelessness and ultimately repetitive thoughts and emotions of bitterness.

It is important that the OCD patients did not suffer from an embitterment syndrome. The PTEDq was here used to assess the emotion of bitterness reflecting all the limitations for the patients induced by the illness. There is no overlap between metacognition and bitterness, but an association between MSQ-30 as a cognitive style and PTEDq as an emotional style.

Societal prejudices and stigmas against mental illnesses are also the product of cultural-societal basic assumptions and thus illnesses such as OCD are associated with, among other things, low intelligence, madness, weakness of will and character, lack of self-discipline or even classified as threatening [25]. Because the OCD patients themselves, as formerly ‘healthy’ people, have grown up with those defamatory basic assumptions towards psychiatric illnesses and have internalized these stigmas, they carry the division of ‘normal’ versus ‘abnormal’ within themselves [26]. This aspect can only be understood as a clear mortification and degradation of the self-image and one’s own existence and consecutively lead to intense bitterness. In this context, it is reported that patients adopt stigmatizing reservations in the form of self-stigmatization, which can lead to a loss of individual self-worth and, above all, self-efficacy [27]. The fact that unfair, unjust treatment of compulsive patients, but also of many other mentally ill persons, is not just pure anticipation on the part of those affected but actually takes place in reality can be proven by numerous studies [28]. This leads to a further explanatory approach to secondary embitterment.

An increased level of bitterness can be explained not only by the fact that the psychiatric illness itself is experienced as an injustice of fate as well as a mortification of the metacognitively distorted self-concept, but also by the fact that compulsive patients are actually confronted with injustice and degradation in everyday life significantly more often than mentally healthy people. In the long term, there is thus an increased likelihood of developing a pathological level of bitterness. A frequently studied and empirically well-documented example of everyday injustice towards people with mental illness is the so-called testimonial injustice [29] According to Fricker’s views [30] testimonial injustice is an ‘unjustified downgrading of a person’s credibility due to a negative prejudice against their social identity’. What sounds quite abstract in theory can be found ubiquitously in daily practice. For example, mentally ill people die, on average, 10–20 years earlier than the general population due to treatable somatic conditions [31]. One of the causal factors for this is assumed to be that people with mental illness are disproportionately often ignored in the somatic care system [32]. Other empirical studies show that practitioners do not trust people with severe psychiatric diagnoses to make participatory decisions [33] or that physical complaints in the emergency room are often causally assigned to the patient’s underlying psychiatric conditions without explicit diagnosis [34].

Furthermore, OCD patients also experience more unfair behaviour in their social life. A study conducted on American adolescents by Storch et al. [35] demonstrated that children and adolescents with OCDs are exposed to a significantly higher rate of bullying and exclusion compared to mentally healthy peers or classmates with diabetes mellitus type 1. In particular, if OCD patients are repeatedly exposed to unfair treatment by others, and in areas of life that are important to them over the course of their lives, and they associate this causally with their mental illness, then this can cause chronic embitterment. This would also explain why OCD patients with early onset of the disease and thus a long disease duration showed a greater degree of bitterness. It can be speculated that these patients were already more frequently confronted with belittling and injustice than OCD patients with a short duration of illness. The study by Muschalla and Kenne [36] also proves the connection between the extent of feelings of bitterness and the number of negative life events.

The fact that the level of the embitterment was associated with general anxiety, depressive symptoms and reduced self-esteem is in line with the results of previous studies [11, 37]. This can be explained by the fact that the embitterment affect is a complex emotion in which feelings of hopelessness, helplessness and fear, among others, are interwoven [4]. The embitterment syndrome is also clinically characterized by self-blame, which could explain the link to reduced self-esteem [5]. The association between the psychosocial functioning level and the degree of clinical impairment with embitterment intensity is also consistent with the findings of Almeida et al. [38] and Gao et al. [11].

The OCD patients in our study who were in a partnership at the time of data collection had significantly lower levels of bitterness than those who were not in a relationship. A happy partnership generally acts as a protective factor for both physical and mental health [39]. It can be assumed that this positive influence is also reflected in the level of pathological bitterness. Mention should also be made of a study by You and Ju [40], which demonstrated that social support reduced feelings of bitterness.

Our study has a number of limitations. First of all, the rather small number of cases in the two group populations should be mentioned. Therefore, the results have to be regarded with cautions in this exploratory study and should be replicated in the future as well as analyzed by using a correction procedure for multiple testing (e.g. Bonferroni). Furthermore, this is a monocentric study of moderately ill and outpatient OCD patients, so it is hardly possible to make general statements about the extent of bitterness in the overall very heterogeneous group of OCD patients. Although the questionnaires used here have been validated, they are not questionnaires routinely used in psychiatric practice. Our study only covers one point in time in the sense of a cross-section; a longitudinal study with possible changes in the degree of bitterness, depending on the course of the disease, would be meaningful and indicated here. Embitterment disorderwas described first by Michael Linden and it is officially not a disorder, but it was discussed within the preparation of DMV 5 and ICD 11. Here, the validated PTEDq was used, in order to assess the emotion of embitterment in OCD patients. The patients did not fulfill the diagnostic criteria for embitterment disorder. Furthermore, it was not possible to investigate clear relationships between embitterment, anxiety, depression and further psychopathological parameters, since OCD patients showed -as usual- a lot of this disorder features.

Our findings on the emotion of embitterment in patients with OCD can be summarized as important indications that this mental disorder is experienced by the affected patients, who are characterized by metacognitive distortions, as an injustice of fate as well as a mortification of their self-image. Due to their mental illness, those affected more frequently experience belittling, rejection or injustice in everyday life. The consequences are a multi-layered and persistent threat to numerous areas of life with which the patient is repeatedly confronted, while at the same time they are often helpless and powerless in the face of the illness. It is not uncommon for them to feel that they can do little to change their illness and life situation, which makes them bitter. The leading effect of bitterness, in turn, has an overall unfavourable effect on the course of the disease and also on the success of the therapy, as it was found for example in patients with rheuma [11] According to Linden’s theory [5], embitterment disorder shows a clear tendency towards chronicity and can lead to a significant restriction of life as well as long periods of incapacity for work, up to complete disability. It is therefore necessary to screen patients with OCD, not only for depressive symptoms or anxiety but also specifically for feelings of embitterment, in order to be able to initiate appropriate psychotherapeutic measures at an early stage.

## Data Availability

Data are easily available on request from the corresxponding author.
